# Risk factors of obstructive sleep apnea among nigerian outpatients

**DOI:** 10.5935/1808-8694.20120029

**Published:** 2015-10-20

**Authors:** Olusola Ayodele Sogebi, Adegboyega Ogunwale

**Affiliations:** a FWACS, FMCORL (Lecturer/Consultant ENT Surgeon); b FWACP (Consultant Physician (Psychiatry))

**Keywords:** nigeria, prevalence, risk factors, sleep apnea, obstructive, snoring

## Abstract

Obstructive sleep apnea (OSA) is a medical condition with adverse consequences. OSA is credited to be a sleep disorder that disproportionately affects blacks. The Berlin Questionnaire (BQ) is a screening questionnaire for OSA.

**Objective:**

To describe the risk factors associated with OSA among adults attending an out-patient specialist clinic. Study design: Prospective, clinical study including adult outpatients attending a specialist clinic.

**Method:**

Data was collected using a questionnaire incorporating the BQ and patients were divided into low and high risks of OSA. The risk factors associated with OSA in the univariate analyses were subjected to a multivariate binary logistic regression model. Adjusted odds ratios with 95% confidence intervals were calculated for these independent variables.

**Results:**

One hundred and ninety five patients participated in the study (Males 56.4%; Age 43.5 ± 15.6 years; Non-habitual snorers 81.5%; High risk OSA 17.4%; BMI 24.1 ± 4.6 kg/m^2^; Obese 12.9%). Six factors including marital status and blood pressure were significantly associated with OSA using bivariate analysis nevertheless age, hours at work, smoking status and BMI remained predictive of OSA on logistic regression analysis.

**Conclusions:**

OSA is common among Nigerian outpatients, may be under-recognized and is associated with risk factors that are amenable to preventive strategies.

## INTRODUCTION

Obstructive sleep apnea (OSA) is a common medical condition with significant adverse consequences^1^ and it is increasingly being recognized as a major source of public health concern in the last two to three decades^2^. Community studies have estimated prevalence rates between 7.5 and 16% in North America^3^, while it is between 3.2% and 4.5% in Asia^4^. It has been found that the risk of OSA increases with possible genetic predisposition; for instance, an association between OSA and the chromosomal region containing the Apolipoprotein E (*ApoE)* gene has been reported^5^. Also a number of proteins including Phosphoinositide 3-kinase, the STAT family of proteins and its related pathways are associated with OSA^6^. OSA is also purported to run in families^7^. Socio-demographic and clinical correlates suggest that it is associated with increasing age i.e. > 30years, the male gender, and chronic nasal congestion^8^. In addition, OSA has been associated with postmenopausal syndrome in women, obesity, hypertension, other cardiovascular diseases^9^ like coronary artery disease, cerebrovascular events, type 2 diabetes, as well as the metabolic syndrome^10^.

The effects of OSA include sleep disturbances manifesting as nighttime and daytime symptoms which include snoring, cessation of breathing and excessive daytime somnolence. Other effects are elevated blood pressure as well as other physical and emotional disturbances. Unfortunately many sufferers of OSA are undiagnosed^11^, ^12^. This is partly because the standard for diagnosis is overnight polysomnography (PSG) which is expensive and not widely available^13^, ^14^. Also, the attendant discomfort of patients spending the night in sleep laboratories render it rather impracticable for large groups of patients whether as a diagnostic tool or as a screening procedure. These considerations are even more compelling in developing resource-poor countries like Nigeria.

A pragmatic solution to the foregoing problems in sleep research has been the development of questionnaires evaluating sleep disorders and OSA. Some of the questionnaires that had been employed include the Epsworth sleepiness scale, ESS, the STOP, and the Cleveland sleep habits questionnaires, CSHQ^15^. However, the Berlin Questionnaire (BQ), remains the most widely used screening questionnaire for obstructive sleep apnea^13^, ^16^. The BQ is a validated tool that assesses the individual's risk of OSA by awarding points based on questions relating to night and daytime symptoms, obesity and hypertension.

Studies done in developed countries to characterize and ascertain the risk factors associated with OSA revealed that some of the risk factors are preventable. OSA is credited to be a sleep disorder that disproportionately affects blacks^17^, and it will therefore be essential to study this phenomenon among a purely black population. A previous study conducted in Nigeria reported that Obstructive Sleep Apnea Syndrome (OSAS) may be more common than initially imagined among Nigerians, and that respondents with a high risk for OSAS were more likely to be obese (BMI > 30 kg/m^2^), had higher mean ESS score, and had chronic medical condition than those who were at lower risk^18^. A limitation of that study was that it did not quantify the risk factors associated with OSA. This present study aims to describe and quantify the risks that are associated with OSA in adult patients attending a specialized clinic in a sub-urban tertiary hospital in Nigeria. The knowledge and magnitude of the risk factors create increased awareness, assist in sensitizing the health care providers to the burden, and could assist in formulating a control programme which would aid in preventing the sequelae of OSA.

## METHOD

Study design: This is a comparative, cross-sectional, hospital based study.

### Study location

The Olabisi Onabanjo University Teaching Hospital is a tertiary referral hospital, which is located in the suburban town of Sagamu in south-western Nigeria, and serves a population of about 250,000 people, constituting patients from the township and other adjourning cities including the state's capital city, and expanding towns overflowing from Lagos, the commercial nerve center of the country. The ENT clinic receives patients referred from other hospitals and other clinics within the hospital. The clinic is run by two ENT surgeons as well as trainee physicians who work under supervision.

### Study participants

Consecutive adult patients aged 18 years and above attending the clinic between February and May 2010 were recruited into the study. Informed consent was obtained from each participant after the general content and purpose of the study had been explained by the investigators.

### Data collection

Data was collected from the participants by OAS and the trainees with the use of a pretested questionnaire structured to incorporate the Berlin (validated) questionnaire. The questionnaire was divided into three sections. Section A sought information on the demography and social habits of the patients. Section B was essentially the Berlin questionnaire which had questions related to snoring, night and daytime symptoms experienced by the patients, presence of high blood pressure, as well as measurement of weight and height. Section C detailed the findings on the general physical, nose, pharyngeal and neck examination. The spouses assisted the patients in answering questions relating to snoring habits and behaviors during sleep. The BMI was calculated as weight in kg divided by height in meter squared i.e. kg/m^2^. Points were allotted to each response to the questions; for instance, 1 point each was allotted for positive response to snoring, and 2 points for positive response to ‘quit breathing during your sleep'. For each of the daytime symptoms, a positive response attracted 1 point, a positive response was also obtained if the participant had a high blood pressure or the BMI was greater than 30kg/m^2^. Participants were classified into High or Low risk of having sleep apnea based on their overall scores in the symptoms categories.

The study protocol was approved by the OOUTH Ethics committee (OOUTH/DA/326/T/11).

### Statistical analysis

Frequency tables were used in describing the demographic and clinical variables. The patients were divided into two groups - low and high risks of OSA. Between-group differences in relation to categorical variables were explored by the use of the Chi square test, while those observed in continuous variables were done using the *Student t*-test. The risk factors associated with OSA at a significance level of *p* < 0.05 in the univariate analyses were then entered into a multivariate binary logistic regression model with each of the associated factors as predictor variables and the risk of OSA (high = 1 *vs*. low = 0) as the outcome variable. Adjusted odds ratios with 95% confidence intervals were calculated for these independent variables. The analysis was done using SPSS version 17.

## RESULTS

One hundred and ninety five patients participated in the study. The socio-demographic characteristics of the respondents are as shown in Table 1. Males constituted 56.4% of the participants. The ages ranged from 20 to 79 years with a mean age of 43.5 ± 15.6 years (mean ± SD). Majority of the patients (76.4%) had minimum of secondary school education, while about 9% did not have formal education. More than half were married at the time of the study. Almost two-thirds of the patients were not consuming alcohol in any form, while one-fifth were irregular alcohol consumers. Slightly over 5% of the participants were current smokers. Most of the patients worked for between 5-8 hours every working day.

**Table 1:** Socio-demographic characteristics of the patients.

Variable	N	%
Sex		
Male	110	56.4
Female	85	43.6
Age range		
20-29	41	21.0
30-39	52	26.7
40-49	39	20.0
50-59	24	12.3
60-69	21	10.8
70-79	18	9.2
Mean +/- SD	43.5 +/- 15.6	
Level of Education		
No formal education	18	9.2
Primary	28	14.4
Secondary	57	29.2
Tertiary	92	47.2
Marital status		
Single	53	27.2
Married	116	59.5
Others: Separated, Divorced, Widow(er)	26	13.3
Consumption of alcohol		
No	125	64.1
Past use Current use	27	13.8
Sometimes	39	20.0
Regularly	4	2.1
Smoking and use of tobacco		
Never	169	86.7
Past use	16	8.2
Current use	10	5.1
Hours at work		
< 5	22	11.3
5-8	101	51.8
> 8	72	36.9

Table 2 shows the clinical characteristics of the patients, with majority of the patients being non-habitual snorers while 17.4% had a high risk of developing obstructive sleep apnea according to the Berlin questionnaire criteria. The BMI of the patients ranged from 15.6 to 37.1 with a mean of 24.1 ± 4.6 kg/m^2^ (mean ± SD). Close to 13% of the patients were obese.

**Table 2:** Clinical characteristics of the patients.

Variable	N	%
Snoring status		
Non-habitual snorer	159	81.5
Habitual snorer	36	18.5
BMI Range		
15.0-19.9	38	19.5
20.0-24.9	84	43.1
25.0-29.9	48	24.6
30.0-34.5	21	10.8
35.0-39.9	4	2.1
Mean +/- SD	24.1 +/- 4.6	
Risk of Obstructive sleep apnea		
Low	161	82.6
High	34	17.4

Table 3 explored the association between the respondents' characteristics and the risk of developing OSA. Age, marital status, hours at work, smoking, blood pressure and BMI were found to be significantly associated with high risk of OSA using bivariate analysis. Further analysis to determine if these variables were independent predictors for high risk of OSA using logistic regression shows that marital status and blood pressure dropped out as independent predictors. Age, hours at work, smoking status and BMI remained predictive of high risk of OSA (Table 4).

**Table 3:** Characteristics in relation to Risk of OSA.

Variable	Risk of OSA		Statistics	*p*
	Low n = 161 (%)	High n = 34 (%)	Statistics	*p*
Sex: Male	92 (57.1)	18 (52.9)	0.202	0.653
Female	69 (42.9)	16 (47.1)		
Age: Mean	41.8 +/- 15.8	51.3 +/- 12.0	3.311^a^	0.001
Level of education				
Nil formal	14 (8.7)	4 (11.8)		
Primary	19 (11.8)	9 (26.5)	5.834	0.120
Above Primary	128 (79.5)	21 (61.8)		
Marital status				
Single	52 (32.3)	1 (2.9)		
Married	89 (55.3)	27 (79.4)	12.254	0.007
Others	20 (12.4)	6 (17.7)		
Work hours: Mean	7.2 +/- 2.3	9.2 +/- 2.7	4.413^a^	< 0.001
Alcohol consumption				
Never	106 (65.8)	19 (55.9)		
Past	20 (12.4)	7 (20.6)	2.887	0.409
Current	35 (21.7)	8 (23.5)		
Smoking				
Never	148 (91.9)	21 (61.8)		
Past	8 (5.0)	8 (23.5)	22.099	< 0.001
Current	5 (3.1)	5 (14.7)		
Blood Pressure				
Normal	153 (95.0)	22 (64.7)	269.943	< 0.001
Elevated	8 (5.0)	12 (35.3)		
BMI: Mean	22.7 +/- 3.4	30.6 +/- 3.9	12.070^a^	< 0.001

^a^ Statistic is *Student t*-test.

**Table 4:** Risk of developing OSA.

Variable	Odds ratio	Intervalo de Confança a 95%	*p*
Age	1.20	1.08-1.34	0.001
Marital status			
Single	1.00 (Reference)	-	-
Married	1.58	0.01-427.47	0.873
Others	12.67	0.03-4851.04	0.403
Work hours	2.43	1.51-3.89	< 0.001
Smoking			
NoPast	1.00 (Reference) 11.60	-1.09-129.35	-0.042
Current	28.67	1.43-576.31	0.028
BMI	1.72	1.33-2.21	< 0.001
Blood Pressure			
Normal	1.00 (Reference)	-	-
High	0.85	0.11-6.76	0.879

## DISCUSSION

This study revealed that 17.4% of adult Nigerian out-patients included in the sample were at a high risk of developing OSA. This finding is similar to that of a comparable study in the country among Primary health care workers which reported a rate of 19.0%^18^. In India, the overall prevalence of OSA in middle aged urban population is 9.3%^19^ while it is in the range of 12.4% in Pakistan^20^. In Europe it was estimated that between 21.5 of Norwegians^16^ and 24.3% of Belgian truck drivers^21^ were at high risk of OSA while in the US population, 26.0% met the Berlin questionnaire criteria for high risk of OSA^22^. This implies that OSA is a common phenomenon among Nigerian hospital patients, although with comparatively lower rates than those reported among Caucasian populations. Redline et al.^23^ however reported that the prevalence among African-Americans, after adjustment for BMI, seems to be approximately equal to and may exceed that among Caucasians while another study had shown a significantly higher incidence of “probable” OSAHS when African-Americans were compared to the Caucasian-Americans in favor of the former^24^. This finding probably underscores the importance of screening out-patients for OSA as a large proportion would have gone undetected because OSA does not appear to be the primary presenting complaint.

The risk factors that were observed were similar to those found in studies conducted in other parts of the world. Some studies^22^, ^25^ had associated ageing with development of OSA, the risk being found to multiply geometrically in the elderly population^22^. In this study it was discovered that for every year increase in age, the risk increases by 20% (OR = 1.20). This increased risk with age has been attributed to age-related anatomical changes in the pharynx which lead to increased upper airway col-lapsibility^26^. The hours of work was also found to be a risk factor for high risk of OSA. Adewole et al.^18^ also related the stress at work among Nigerians and found a significantly increased risk of OSA among respondents that were involved in stressful than those involved in less stressful jobs in developing OSA. This study corroborates the findings of the earlier one, but also suggests that the average hours at work may be related to the stress experienced at the workplace. Among Belgian truck drivers unrealistic work schedule, (> 50hrs of driving per week), was significantly associated (OR 2.85) with poor sleep quality^21^.

Both past and current smokers were at a significantly higher risk of OSA compared with patients who never smoked. Smoking has been found to cause chronic irritation and inflammation of the airways which can become irreversible. The attendant changes in both anatomical and physiological properties of the respiratory tract significantly affect the airflow with consequent reduction in the quality of sleep. Thus current smoking was found to be an independent correlate (OR 1.75) of daytime sleepiness among Belgian truck drivers^21^, associated positively with difficulty in maintaining sleep among Chinese adolescents^27^, and related to various subtypes of sleep disturbances among the Japanese^28^. Furthermore, exposure to passive smoking at work was associated with short sleep duration among Japanese working men^28^.

In concordance with other studies^19^, ^22^, ^29^, increased BMI has been found to be associated with a high risk of OSA in this study. Obesity has been particularly found to increase the risk of OSA and this study has revealed that in adult Nigerian patients, the risk increases by about 72% (OR = 1.72) for every unit rise in the BMI. Obesity can cause airway narrowing as a result of excess of fat tissue around the neck. Neck circumference corrected for height has also been reported to be useful as a predictor of OSAS^30^. In a study by Pretto et al.^31^ among Australian adults, it was reported that the severity of sleep disordered breathing (SDB) increased progressively over two decades with a yearly increase in median BMI of 0.15 kg/m^2^ for men and 0.14 kg/m^2^ for women thus supporting the assertion that worsening severity in SDB could be primarily attributable to increasing obesity. Probably the most important implication of association of OSA and obesity is hypertension and other cardiovascular sequalae^32^. It has been suggested that OSA should be considered in the differential diagnosis of hypertensive patients who are obese^33^.

This study did find any risk associated with gender. Some studies^2^, ^7^ had reported associations of OSA with the male gender. The male predisposition has been attributed to some factors including inherent structural and functional differences in upper airway during sleep between men and women with more favorable airway mechanics in women^34^, more fat deposition around the pharyngeal airway in men than in women, as well as hormonal differences. Other studies had however reported findings at variance with this information. For instance, among the Saudis, sleep symptoms including witnessed apnea, and excessive daytime sleepiness did not show any statistical difference between the two genders^35^, while among young and middle-aged Pakistanis, male gender was not a significant risk factor for SDB symptoms^36^. Similarly when the PSG finding among Turkish patients were considered, there was no statistically significant difference between the male and female patients regarding the severity of OSAS^37^. Moreover, a study done in the USA to examine the effect of obesity on pharyngeal size in both men and women found there was no correlation between BMI and pharyngeal size in either gender^38^. Other possibilities for this apparent difference may include referral bias and under appreciation of this disease in women by physicians and patients alike^37^. On the other hand, gender differences in clinical presentations may have resulted in more males than females being diagnosed^39^. It seems there may be other factors inherent in the individual that will determine the occurrence, and the severity of OSA other than gender alone. Further work is needed to understand this gender gap in the diagnosis of obstructive sleep apnea^38^. Despite being found to be associated with high risk of OSA, marital status and elevated blood pressure were not found to be independent risk factors for OSA.

The reality that both the diagnosis and treatment of OSA are uncomfortable and relatively unavailable justifies the probing for the risk factors that may be responsible for this phenomenon. Incidentally, some of the risk factors found in this study are preventable or avoidable. Preventive measures will involve adjustments in way of life like avoidance of sedentary lifestyle and being physically active with advancing age^40^. Other possible preventive strategies that can be conveniently deployed in an environment with limited capability for diagnosis and treatment of a condition with serious public health concern and implications include regulation of work hours, control of weight and abstinence from smoking. The value of regular exercise within this preventative context cannot be overemphasized.

This study has some important limitations which must be addressed. The Berlin questionnaire has been a widely used screening questionnaire for OSA but has its constraints. The questionnaire depends on spouse's report, it is prone to recall bias, and its cut off points might not be valid for this population. Validation studies in this environment will be important in this respect. Overnight PSG which is the best method for confirming OSA is currently unavailable in our country and hence the resort to this validated questionnaire. Despite the aforementioned limitations, a previous sleep study in Nigeria had been successfully conducted using this useful instrument^18^. The hospital-based nature of this study limits the gen-eralisability of its findings. The modest sample size and the inability to determine the direction of causality in a cross-sectional study are also limitations. In spite of these limitations however, this study has been able to find the likely risk factors associated with OSA in our environment and could provide a template on which further similar research could be laid.

## CONCLUSION

This study has revealed that OSA is common among Nigerian outpatients, may be under-recognized and is associated with risk factors that are amenable to preventive strategies. It stresses the need for health care providers to adopt structured clinical interview techniques in order to aid its diagnosis and also emphasizes their responsibility to provide the society with education on the necessary life-style modifications which may act as potent means of preventing the disorder.
